# Induction of the differentiation of porcine bone marrow mesenchymal stem cells into premature hepatocyte-like cells in an indirect coculture system with primary hepatocytes

**DOI:** 10.1080/19768354.2020.1823473

**Published:** 2020-10-04

**Authors:** Imran Ullah, Kangmin Seo, Hayeon Wi, Youngim Kim, Seunghoon Lee, Sun A Ock

**Affiliations:** a Animal Biotechnology Division, National Institute of Animal Science, Rural Development Administration, Wanju-gun, Republic of Korea; b Department of Biochemistry, Quaid-i-Azam University, Islamabad, Pakistan

**Keywords:** Mesenchymal stem cells, bone marrow mesenchymal stem cells, hepatocyte-like cells, coculture, Transwell system

## Abstract

Liver transplantation is currently the only option for patients with end-stage liver disease. Thus, other alternate therapeutic strategies are needed. Bone marrow mesenchymal stem cells (BM-MSCs) are nonhematopoietic cells present in the bone marrow stroma that serve as precursors cells for various other cells. In this study, we evaluated the differentiation of porcine BM-MSCs into hepatocyte-like cells using three types of culture systems: hepatic induction medium (HIM), HIM/primary hepatocyte culture supernatant (HCS; 1:1 ratio), and a hepatocyte coculture system (HCCS; primary hepatocytes in the upper chamber, and BM-MSCs in the lower chamber). Primary hepatocytes were isolated from anesthetized healthy 1-month-old pigs by enzymatic digestion. Hepatic-specific marker expression (albumin [*ALB*], transferrin [*TF*], α-fetoprotein [*AFP*]), glycogen storage, low-density lipoprotein, and indocyanine green uptake were evaluated. Upregulation of hepatic-specific markers (*ALB*, *TF*, and *AFP*) was observed by real-time polymerase chain reaction in the HCCS group. Periodic acid-Schiff staining revealed enhanced glycogen storage in hepatocyte-like cells from the HCCS group compared with that from the HIM/HCS group. Furthermore, hepatocyte like-cells in the HCCS group showed improved LDL and ICG uptake than those in the other groups. Overall, our current study revealed that indirect coculture of primary hepatocytes and BM-MSCs enhanced the differentiation efficacy of BM-MSCs into hepatocyte-like cells by unknown useful soluble factors, including paracrine factors.

## Introduction

Liver transplantation is the only option for patients with end-stage liver diseases. However, this strategy is not viable for many patients with liver disorders owing the limited supply of organs (Pinter et al. 2016; Artur et al. 2017). Furthermore, many patients do not qualify for transplantation because of drug abuse, active alcoholism, metastatic cancer, or other medical problems. Because of these limitations, most patients awaiting liver transplantation are unable to receive this treatment (Lee 1993; Sundback and Vacanti 2000). Accordingly, researchers have continued to explore alternate options for patients with end-stage liver disease.

Stem cell-based generation of hepatocyte-like cells may be one such alternative to liver transplantation (Colmenero and Sancho-Bru 2017). Moreover, stem cell-based hepatocyte-like cells were widely used in the fields of regenerative medicine, disease modeling, and drug development (Cherry and Daley 2012; Papanikolaou et al. 2017). Mesenchymal stem cells (MSCs) are adult stem cells that are a rich source of proliferating stem cells and can be isolated from various tissues, including bone marrow, umbilical cord blood, skin, dental pulp, and adipose tissues (Kawashima 2012; Ullah et al. 2015; Ullah et al. 2016; Phinney and Pittenger 2017). Bone marrow (BM)-MSCs are nonhematopoietic cells occurring in the bone marrow stroma that serve as precursors for various other cells (Caplan 2007; Chamberlain et al. 2007). Furthermore, BM-MSCs can be obtained in high quantities without losing their stemness and can be differentiated into hepatocyte-like cells displaying the characteristics of primary hepatocytes (PHs) (Cipriano et al. 2017; Yu et al. 2018; Bandi et al. 2019). The generation of hepatocyte-like cells from BM-MSCs is a compelling concept that has attracted much attention in the field of liver transplantation (Krause et al. 2001). Regardless of published reports on the differentiation of BM-MSCs into hepatocytes, only a limited number of small molecules and factors are known to be involved in the transdifferentiation process (Shimomura et al. 2007; Zemel et al. 2009).

Hepatocyte culture conditions, which should mimic the native physiological niche in which hepatocytes are found, is one of the most commonly used approaches to enhance the differentiation capability of BM-MSCs. Previously, researchers have used in vitro coculture systems with damaged slices of liver or culture in medium conditioned using nonparenchymal liver cells to exploit unknown and essential growth factors derived from liver tissue (Kang et al. 2004; Poll et al. 2006; Soto-Gutiérrez et al. 2007).

In this study, we investigated the potential of porcine BM-MSCs to differentiate into hepatocyte-like cells using stepwise chemical hepatocyte induction medium and exposure to hepatocyte-derived soluble factors by applying condition medium (hepatocyte culture supernatants) and a Transwell coculture system.

## Materials and methods

### Reagents and media

Unless otherwise specified, all chemicals were purchased from Sigma-Aldrich Corporation (St. Louis, MO, USA), and cell culture media were obtained from Gibco (Life Technologies, Carlsbad, CA, USA).

### Animal experiment

All animal experiments were carried out in accordance with the Guide for the Care and Use of Laboratory Animals of our institution and were approved by the Animal Ethics Committee of the National Institute of Animal Science, Rural Development Association, Republic of Korea (approval no. NIAS2015-790).

### Isolation of BM-MSCs from pigs

BM-MSCs were isolated as described previously (Ock et al. 2010). After isolation, BM-MSCs were cultured in Advanced Dulbecco’s modified Eagle’s medium (ADMEM) supplemented with 10% fetal bovine serum (FBS), 1× GlutaMAX (Gibco), and 1% penicillin–streptomycin (10,000 IU and 10,000 µg/mL) at 37°C and 5% CO_2_ in a humidified incubator. After reaching confluence, cells were subcultured for further analyses.

### Harvest of PHs from pigs

Isolation of PHs was performed using a modified two-step enzymatic digestion method in anesthized pigs (Selgan 1976; Dunn et al. 1989). First, the liver was perfused with liver perfusion medium supplemented with 5% FBS through the portal vein. Second, dissociation of hepatocytes was performed by perfusion using liver digest medium at 37°C. The extracted liver was minced, dissociated using a tissue grinder (Chemglass Life Science LLC, Vineland, NJ, USA), and filtered using 100- and 40-μm cell strainers. Recovered hepatocytes were washed twice with hepatocyte wash medium and cultured with hepatocyte maintenance medium (HMM; Williams’ E medium supplemented with 10% FBS and hepatocyte maintenance supplements [0.1 μM dexamethasone and cell maintenance cocktail]). For the next experiment, the concentration of PHs was adjusted (1 × 10^7^ cells/mL), and cryopreservation was performed using Cryo-gold (Revive Organtech, Inc., Irvine, CA, USA) according to the manufacturer’s instructions. PHs with or without freezing and thawing were cultured with HMM for 7 days to analyze hepatocyte function and cell death-related genes.

### Immunofluorescence staining of PHs

After confirmation of the morphology of PHs, cells were cultured on 2-well collagen-coated chamber slides for 1 d, fixed with 4% formalin, and washed three times in phosphate-buffered saline (PBS) containing 3% bovine serum albumin (BSA). The samples were stained with primary antibodies targeting E-cadherin (1:50; Santa Cruz Biotechnology, Japan) for 1 h, washed three times with PBS containing 3% BSA, and incubated with secondary antibody (donkey anti-goat IgG-FITC; 1:100; Santa Cruz Biotechnology) for 1 h. Cells were counterstained with 1 μg/mL propidium iodide (PI) containing RNase for 30 min and mounted with Vectashield (Vector Laboratories, Inc., Burlingame, CA, USA). The slides were observed under a fluorescence microscope.

### Generation of BM-MSC-induced hepatocyte-like cells

**Figure 1.**
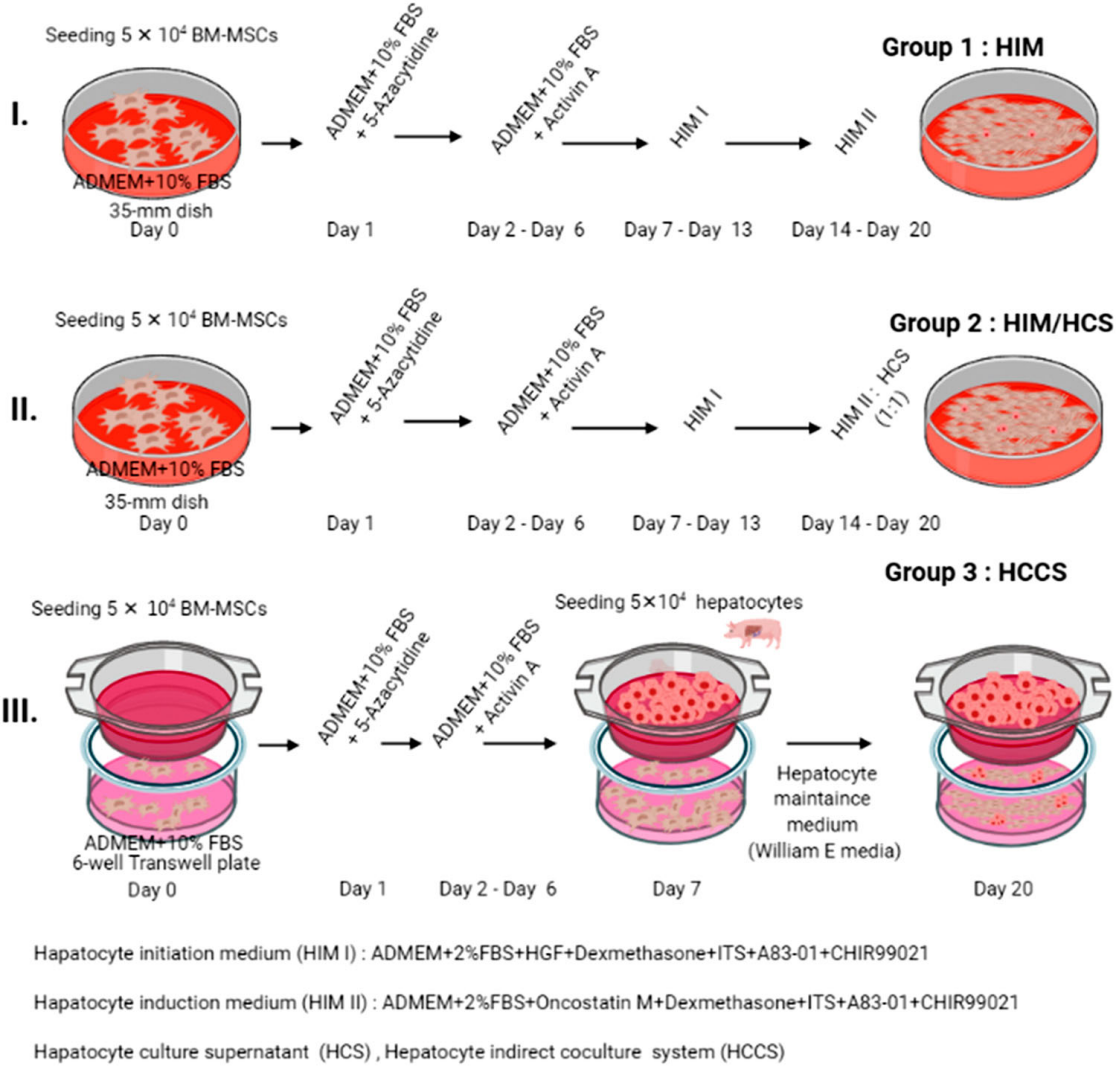
Strategies for differentiation of BM-MSCs into hepatocyte-like cells. First, 5 × 10^4^ BM-MSCs were seeded on 35-mm dishes (day 0) and treated with 1 μM 5- Azacytidine on day 1. Cells were then induced using different methods until day 20. All groups were treated with 30 ng/mL Activin A for 24 h on day 3. Furthermore, BM-MSCs were induced into hepatocyte-like cells using stepwise chemical induction medium, such hepatocyte initiation medium (HIM I) and hepatocyte induction medium (HIM II) (group I, HIM) or a mixture (1:1) of HIM and hepatocyte culture supernatant (HCS) (group II, HIM/HCS). HIM/HCS was exchanged with fresh medium after every 3 days. In group III, BM-MSCs were indirectly cultured with hepatocytes in a coculture system (HCCS) (group III, HCCS) using Transwells. The concentration of hepatocytes was the same as that of BM-MSCs. Hepatocytes were exchanged every week.

For the generation of BM-MSC-induced hepatocyte-like cells, 5 × 10^4^ BM-MSCs were seeded in 6-well collagen-coated dishes in ADMEM (Gibco). After 24 h, cells were treated with 1 μM 5-azacytidine (5 Aza) and were kept for next 24 h in ADMEM supplemented with 10% FBS. This was followed by treatment with Activin A (30 ng/mL) for 5 days. The cells were then replaced with hepatic induction medium I (HIM I: ADMEM, 2% FBS, 20 ng/mL hepatocyte growth factor [HGF], 2 µM A83-01, 3 µM ChiR99021) for 7 days. Furthermore, cells were treated with hepatic induction medium II (HIM II: ADMEM, 2% FBS, 10 ng/mL Oncostatin M, 0.1 µM dexamethasone, 1 × ITS, 2 µM A83-01, 3 µM ChiR99021) for the next 7 days (group 1), as shown Figure 1. In group 2, on day 14, HIM II and primary hepatocyte culture supernatant mixture (HCS) were used at a ratio of 1:1. HCS were collected by culturing PHs (2 × 10^6^ cells) in a 25 T culture flask with 6 mL HMM for 3 days, and supernatants were collected. The supernatants were filtered with a 0.2-μm syringe filter, divided into 1-mL aliquots, and stored in a –70°C deep freezer for further use. In group 3, an HCCS was used in which 5 × 10^4^ BM-MSCs were seeded in the bottom chambers of a 6-well Transwell dish (Nunc 140663; Roskilde, Denmark) in ADMEM and treated with 1 μM 5 Aza for 24 h, followed by treatment with 30 ng/mL activin A for 24 h (Figure 1). Next, 2 × 10^5^ porcine PHs were seeded in the upper Transwell cell culture inserts. HMM was added to the 6-well Transwell dish, and cells were monitored for 21 days. PHs were replaced with new PHs every week.

### Reverse transcription quantitative polymerase chain reaction (RT-qPCR)

Total RNA was isolated from cells using an RNeasy mini kit (Qiagen GmbH, Hilden, Germany) and quantified with a spectrophotometer (NanoDrop 1000; Thermo Scientific, Wilmington, DE, USA). cDNA was synthesized from total purified RNA (1 μg) using an Omniscript reverse transcription kit (Qiagen) with 10 × RT buffer, dNTP mix, RNase inhibitor, and 10 μM OligodT primer at 37°C for 1 h. RT-qPCR was performed using a StepOnePlus Real-Time PCR System (Applied Biosystems, CA, USA) with SYBR Green master mix (Thermo Fisher Scientific) supplemented with 10 μM specific primers (Supplementary Table 1). Specific primer sets were designed using Primer express software version 3.0.1 (Applied Biosystems). All experiments were carried out in triplicate, and the glyceraldehyde 3-phosphate dehydrogenase gene was used as an internal control. Relative quantification of gene expression was calculated as the relative quantity (RQ) value after calculating ΔCt and ΔΔCt values.

### Periodic acid-Schiff (PAS) staining of hepatocyte-like cells

Hepatocyte-like cells were evaluated to assess their glycogen storage ability using PAS staining. Briefly, hepatocyte-like cells were fixed with 4% paraformaldehyde for 30 min, treated with oxidizing agent (1% periodic acid solution) for 5 min at room temperature, washed three times with distilled water, and treated with Schiff’s reagent for 15 min at room temperature. Finally, cells were rinsed with distilled water, counterstained with Mayer’s hematoxylin for 1 min, and washed with distilled water. Glycogen storage was observed under a light microscope.

### Indocyanine green (ICG) assay

For the ICG assays, ICG solution was added to hepatocyte-like cells at a final concentration of 1 mg/mL, and cells were incubated for 1 h at 37°C. After 1 h, cells were washed with DPBS and observed under a microscope to evaluate ICG uptake. To induce ICG release, cells were incubated in culture medium without ICG solution for 6 h at 37°C. After 6 h, cells were observed under an inverted microscope for ICG release.

### Low-density lipoprotein (LDL) uptake assay

For analysis of LDL uptake, cultured cells were incubated with 10 μg/mL Dil-Ac-LDL (acetylated LDL labeled with 1,1′-dioctadecyl-3,3,3′-tetramethylindo-carbocyanine perchlorate; Invitrogen, Carlsbad, CA, USA) for 3 h at 37°C, counterstained with 1 μg/mL DAPI for 30 min, and observed under a fluorescence microscope.

### Statistical analysis

Data were analyzed by one-way analysis of variance and post-hoc Tukey tests with IBM SPSS statistics 24. PCR data were expressed as RQ, and error bars represent RQ ± min and RQ ± max. Differences with *p* values of less than 0.05 were considered significant. All experiments were performed in triplicate.

## Results

### PH isolation and characterization

**Figure 2.**
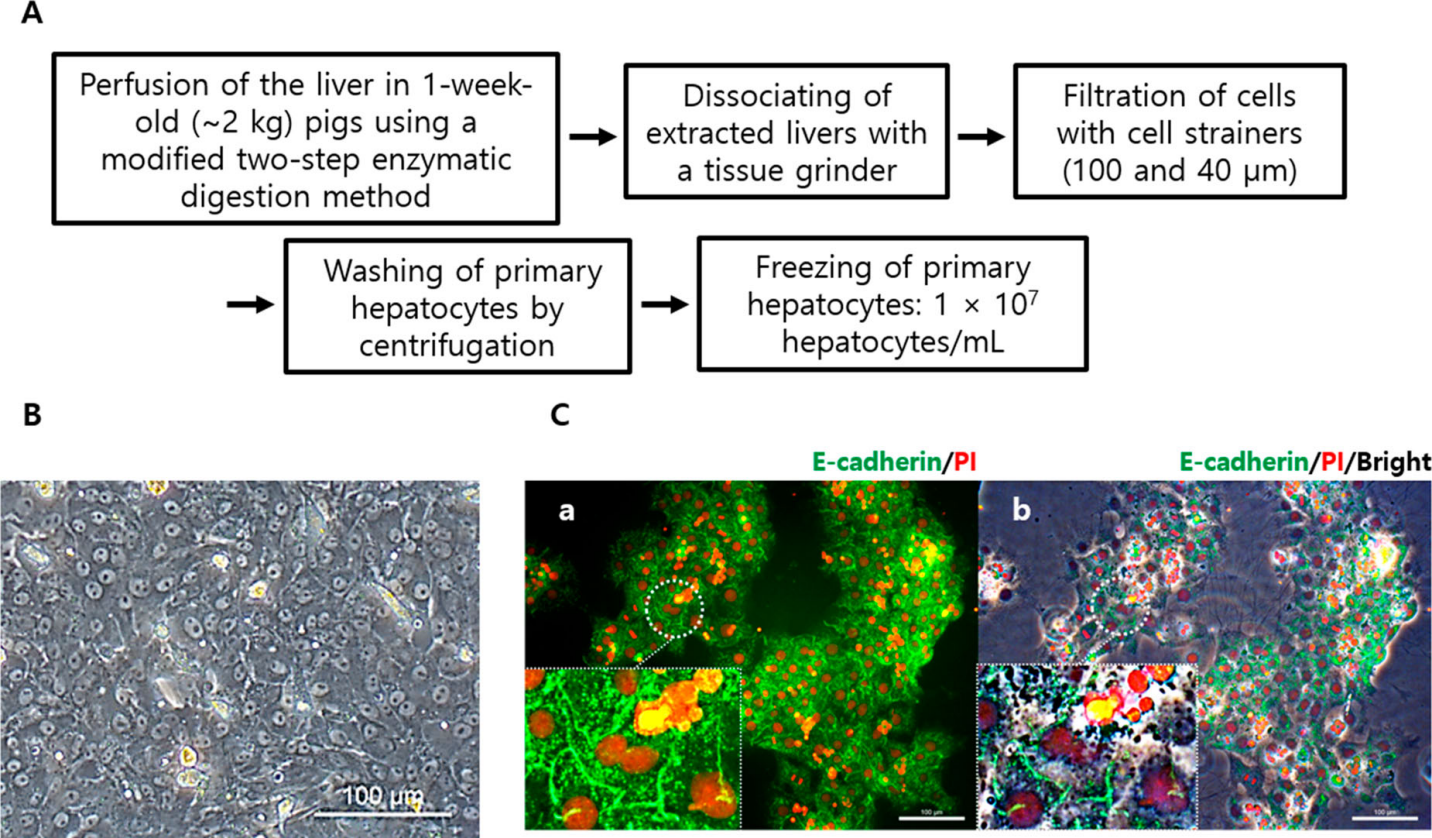
Isolation and confirmation of porcine primary hepatocytes. (A) Livers of anesthetized 1-week-old pigs were perfused with liver perfusion medium containing 5% FBS and liver digest medium at 37°C. The extracted liver was minced, dissociated with a tissue grinder, filtered using 100 and 40 μm cell strainers, washed with hepatocyte wash medium, and kept in LN_2_ after cryopreservation (1 × 10^7^ cells). (B) Fresh porcine primary hepatocytes showed both polygonal cytoplasm and binuclear hepatocytes. (C) Binuclear hepatocytes were observed using anti-E-cadherin antibodies and PI staining (white dot circle and magnified boxes). a and b showed merged images of E-cadherin (green)/PI (red) and E-cadherin (green)/PI (red)/bright field, respectively.

PHs were successfully harvested using a modified two-step enzymatic digestion methods and showed both polygonal cytoplasm and binuclear hepatocytes characterized by anti-E-cadherin antibody reactivity and PI staining (Figure 2).

**Figure 3.**
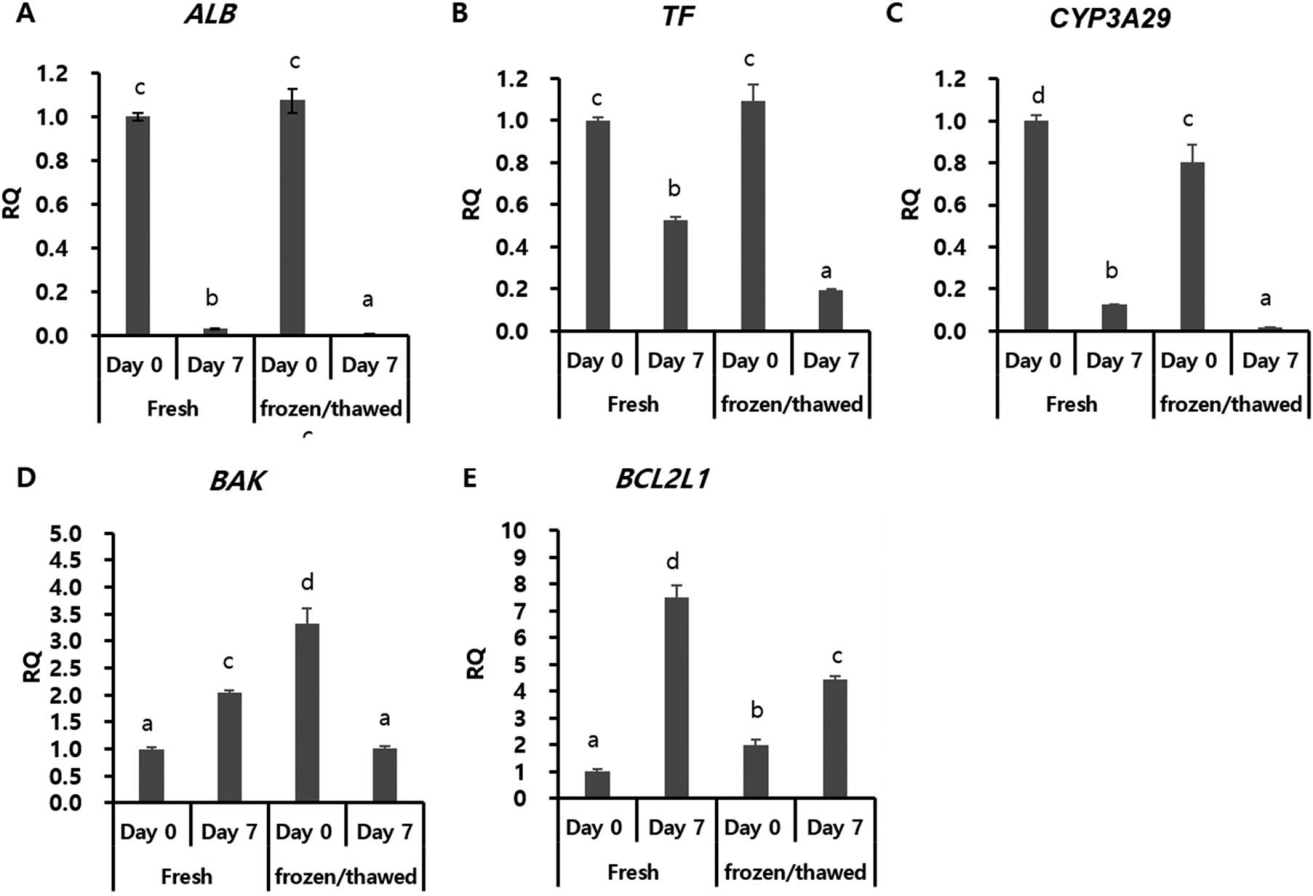
Expression of hepatocyte-specific and apoptosis-related genes in porcine primary hepatocytes cultured in vitro for 1 week. Fresh and frozen/thawed hepatocytes were cultured in hepatocyte maintenance medium (HMM) for 7 days. Fresh hepatocytes without in vitro culture were used as a control for each group. A, B, and C show albumin (*ALB*), transferrin (*TF*), and *CYP3A29*, respectively. D and E show the pro-apoptotic gene *BAX* and the anti-apoptotic gene *BCL2L1*, respectively. RQ: relative quantification; error bars represent RQ ± min and RQ ± max; differences were considered significant at *p *< 0.05. All experiments were performed in triplicate. Letters a, b, c, and d indicate significant differences (*p* < 0.05) among groups.

Without culturing on day 0, there were no significant differences in *ALB* and *TF* expression between fresh PHs and frozen/thawed PHs. However, there were slight differences in *CYP3A29* expression (fresh: frozen/thawed, 1:0.8). Regardless of the freeze/thaw process, when PHs were cultured in vitro for 7 days, PHs showed major changes in liver function-related genes; for example, *ALB* (fresh: frozen/thawed, 0.03:0.01), TF (fresh: frozen/thawed, 0.53:0.19), and *CYP3A29* (fresh: frozen/thawed, 0.13:0.02) expression levels were significantly decreased (Figure 3(A–C)). Among groups, frozen/thawed PHs at day 7 showed the lowest gene expression for all three genes.

After in vitro cultivation of PHs for 7 days, fresh PHs showed upregulation of both pro-apoptotic (*BAK*) and anti-apoptotic (*BCL2L1*) genes, and frozen/thawed PHs showed decreased expression of *BAK* and increased expression of *BCL2L1* (Figure 3(D–E)).

Based on these findings, further studies were performed with frozen/thawed PHs to maintain the same conditions.

### Morphological changes during the differentiation of BM-MSCs into hepatocyte-like cells

**Figure 4.**
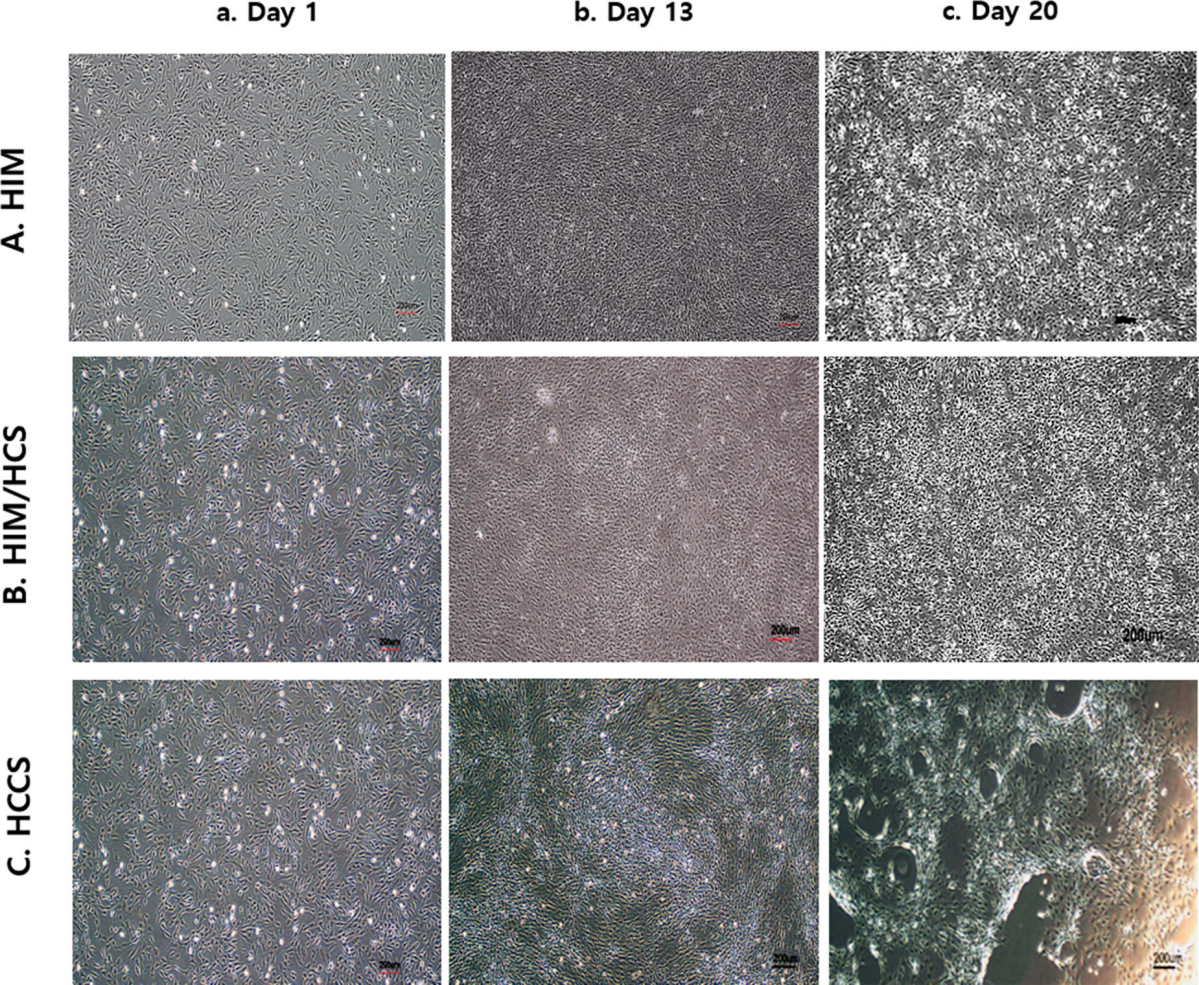
Morphological transition of BM-MSCs into hepatocyte-like cells after 3 weeks. Each group was induced for 21 days and observed under a microscope. A, B, and C show HIM, HIM/HCS, and HCCS groups, respectively. a, b, and c show induction time intervals (day 1, day 13, and day 20, respectively). Scale bars = 200 µm.

BM-MSCs were induced into hepatocyte-like cells and underwent major morphological transitions (Figure 4). The BM-MSCs exhibited a round morphology after 5-Aza treatment (Figure 4(a)) and were continuously transformed upon HIM I (Figure 4(Ab)), HIM II (Figure 4(Ac)), and HIM II + HCS treatment (Figure 4(Bc)). However, clear clusters composed of hepatocyte-like cells were not found in the HIM or HIM/HCS groups. Furthermore, the HCCS group, including the Transwell coculture system with PHs, slowly transformed the cells into round clustered hepatocyte-like cells (Figure 4(Cc)) after 21 days.

### Gene expression of major hepatic function markers

**Figure 5.**
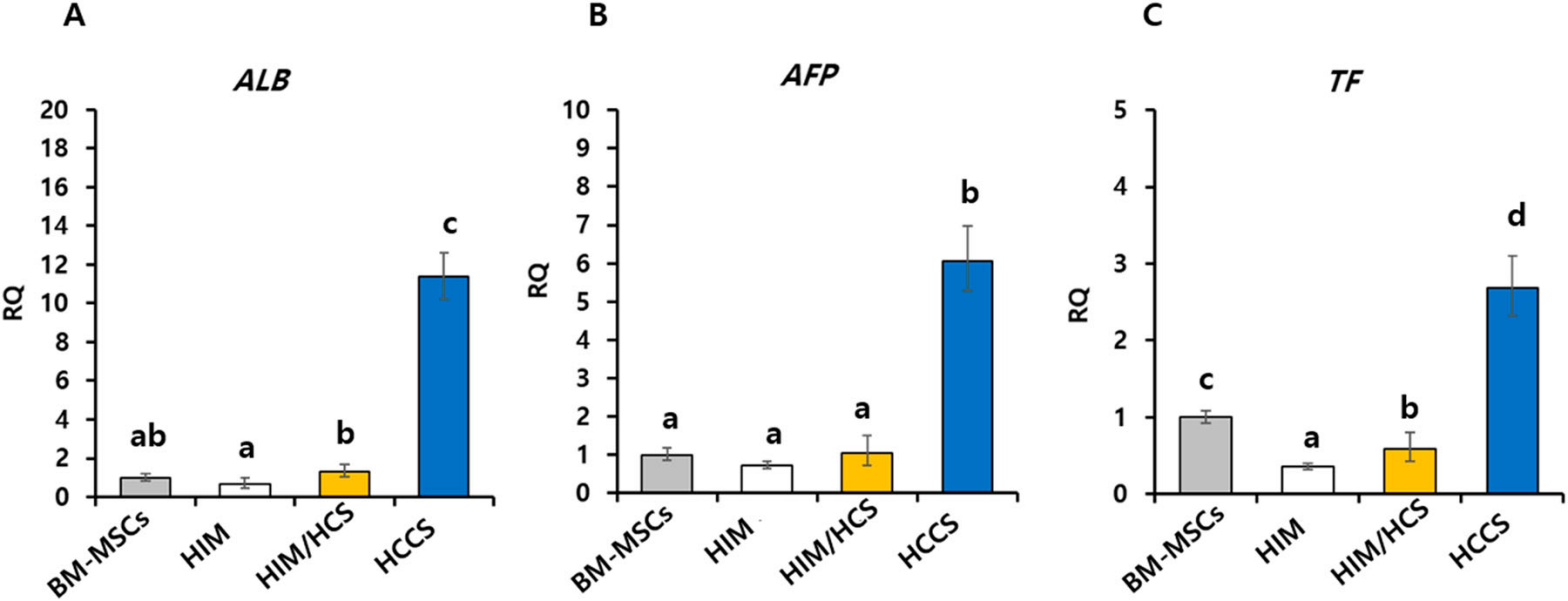
Expression of hepatic-specific markers in hepatocyte-like cells induced from porcine BM-MSCs. The hepatic-specific markers (A) *ALB*, (B) *AFP*, and (C) *TF* were analyzed in HIM, HIM/HCS, and HCCS groups. RQ: relative quantification; error bars represent RQ ± min and RQ ± max; differences were considered significant at *p* < 0.05. All experiments were performed in triplicate. Letters a, b, c, and d showed significant differences (*p* < 0.05) among groups.

To confirm degree of induction of hepatocyte-like cells, major hepatic markers were analyzed by RT-qPCR (Figure 5). The expression of *ALB* was significantly higher in the HCCS group (11-fold) compared with that in BM-MSCs in the control group (Figure 5(A)). Furthermore, we observed upregulation of *AFP* (6.06-fold, Figure 5(B)) and *TF* (2.68-fold, Figure 5(C)) in the HCCS group compared with that in the control group. These gene expression data showed that the HCCS was better than the other treatments and could reinforce the ability of BM-MSCs to differentiate into hepatocyte-like cells.

### ICG uptake and release of hepatocyte-like cells

**Figure 6.**
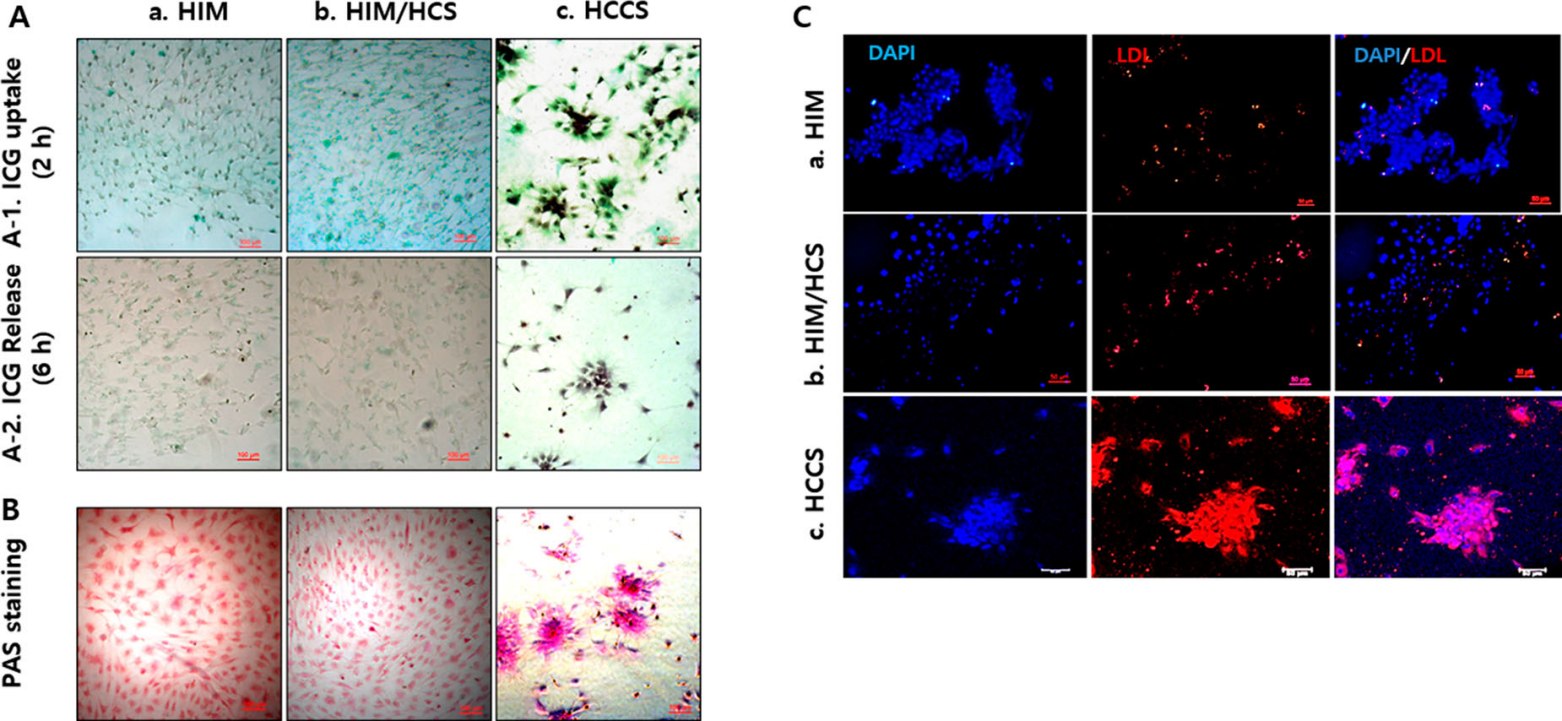
Hepatocyte function assays in hepatocyte-like cells induced from porcine BM-MSCs. (A) ICG uptake and release assays were performed for evaluation of toxic agent absorption and degrading ability. Uptake tests were performed in hepatocyte-like cells after treatment with ICG for 2 h (A-1), and release tests were performed at 6 h after treatment (A-2). Scale bars = 100 µm. (B) Glycogen storage was analyzed by PAS staining (red). Scale bars = 100 µm. (C) The LDL uptake tests in hepatocyte-like cells. Red fluorescence: LDL; blue fluorescence: DAPI (nuclear staining). Scale bar = 50 µm. a, b, and c represent HIM, HIM/HCS, and HCCS groups, respectively.

Hepatocyte-like cells possess numerous functions of mature hepatocytes. We performed ICG uptake and release assays. Hepatocyte-like cells in all groups showed uptake of ICG after 2 h of treatment (Figure 6). In particular, the HCCS group showed more prominent absorption of ICG compared with the other groups (Figure 6(A-1c)). Culture of hepatocyte-like cells in their respective media without ICG for 6 h resulted in partial release of ICG in all groups (Figure 6(A-2)). Increased release of ICG was observed in the HCCS group compared with that in the HIM and HIM II/HCS groups.

### LDL uptake and PAS staining analysis

All three groups showed positive reaction by PAS staining, indicating glycogen accumulation (Figure 6(B)). The HCCS induced hepatocyte-like cells that stained more strongly than those in the other two groups. Moreover, clusters composed of hepatocyte-like cells showed darker staining than unclustered cells in the HCCS group. Hepatocyte-like cells were also analyzed for LDL uptake (Figure 6(C)). Notably, the hepatocyte-like cells in the HIM II, HIM II/HCS, and HCCS groups showed LDL uptake with positive nuclear staining. The HCCS induced hepatocyte-like cells with higher LDL uptake than in the other groups (Figure 6(Cc)). Additionally, clusters composed of hepatocyte-like cells in HCCS showed stronger staining than unclustered cells, indicating increased LDL uptake.

## Discussion

In this study, three methods were evaluated for differentiation of BM-MSCs into hepatocyte-like cells based on morphological changes, functional similarities to hepatocytes, and expression of hepatic-specific markers. The use of BM-MSCs for the generation of hepatocyte-like cells is important with regard to the availability of sustainable, readily available donor cells for liver diseases. Previous reports have shown that BM-MSCs can differentiate into hepatocyte-like cells by modulation of cell culture conditions (Lee et al. 2004; Shimomura et al. 2007). However, the low differentiation potential of BM-MSCs into hepatocyte-like cells has made it difficult to use this approach for clinical applications; accordingly, further investigations are required to increase the efficacy and repeatability of hepatocyte-like cell differentiation from BM-MSCs.

After 3 weeks of induction into hepatocyte-like cells, all experimental groups showed morphological transitions to cuboidal or polygonal shapes, as previously reported (Prasajak and Leeanansaksiri 2013; Patil et al. 2014). However, because of cellular stimulation with exogenous cytokines, analysis of morphological changes only cannot confirm differentiation success. To date, different protocols have been used for generation of hepatocyte-like cells, varying from the selection of exogenous cytokines, culture media, and induction intervals to analysis of the expression of a specific set of markers for differentiation efficacy (Kamiya et al. 1999; Baharvand et al. 2006; Kim et al. 2011). In this study, we compared differentiation protocols, including step-wise chemical induction with growth factors and small molecules and exposure of BM-MSCs to hepatocyte-derived soluble growth factors. We aimed to explore the four-step differentiation of BM-MSCs, i.e. epigenetic reprograming, endoderm induction, hepatocyte differentiation, and maturation, following the addition of small molecules to hepatocyte-like cells. We showed that exposure of BM-MSCs to 5-Aza improved reprograming efficiency to a pluripotent state through epigenetic modifications, such as binding to DNA methyltransferase (Mikkelsen et al. 2008; Pennarossa et al. 2013). Next, activin A, which belongs to the transforming growth factor (TGF)-β family, was used to convert mesoderm cells to endoderm cells using a dose-dependent approach (D'Amour et al. 2005). Finally, HGF (endoderm specification) and Oncostatin M (interleukin-6 family cytokine) were supplemented for hepatocyte differentiation (Schmidt et al. 1995; Kinoshita et al. 1999; Lee et al. 2004). Interestingly, the small molecule A83-01 further promotes the hepatic differentiation process by acting as a TGF-β activating receptor-like kinase inhibitor and blocking the epithelial-to-mesenchymal transition pathway (Tojo et al. 2005). Additionally, CHIR99021 is an aminopyrimidine derivative that is a powerful inhibitor of GSK3 and acts as an inhibitor of the WNT pathway to promote conversion efficiency from somatic cells to hepatocyte-like cells (Lim et al. 2016). These two small molecules can act not only as activators of *C-MYC* and *KLF4* genes but also as promoters of the mesenchymal-to-epithelial transition (Lim et al. 2016). However, in our study, we did not observe upregulation of hepatocyte function-related genes (*ALB*, *TF*, and *AFP*) in BM-MSC-derived hepatocyte-like cells induced using step-wise chemical induction media for 3 weeks. These results contradicted a previous report of long-term differentiation (6 weeks) (Lee et al. 2004). These different results may be related to the variation in hepatocyte differentiation time because the previous report showed that BM-MSC-derived hepatocyte-like cells were not detected until 2 weeks and showed positive expression after 4 weeks (Lee et al. 2004). In our study, hepatocyte function-related assays, such as PAS staining, LDL uptake assays, and ICG uptake assays, were performed at 3 weeks, and our protocol may have enhanced early differentiation of BM-MSCs into hepatocyte-like cells.

The Transwell coculture of BM-MSCs with PHs may enhance hepatocyte differentiation by BM-MSCs and increase the proliferative activity of PHs (Mizuguchi et al. 2001; Deng et al. 2008). Many reports have shown that stem cells characteristics are mostly regulated by the cells and secreted cytokines constituting the extracellular environment of the stem cells (Li and Xie 2005; Moore and Lemischka 2006). These results further supported that stem cells undergo reprograming or transdifferentiation into host organ parenchymal cells after transplantation into a new microenvironment. Taken together, these previous reports support our hypothesis that Transwell cultures of BM-MSCs with PHs boost the hepatic differentiation ability of BM-MSCs, as demonstrated by higher glycogen and LDL uptake and increased expression of hepatic-specific markers. In our Transwell culture system, the BM-MSCs were not in direct contact with the PHs, implying that BM-MSC differentiation was achieved via soluble factors, including paracrine factors secreted by PHs, as previously reported (Poll et al. 2006; Soto-Gutiérrez et al. 2007). Accordingly, we concluded that HCS could not improve hepatocyte differentiation of BM-MSCs because of a lack of sufficient paracrine effects/factors.

In conclusion, our current study demonstrated that the PHs could enhance the differentiation efficacy of BM-MSCs into hepatocyte-like cells through exchange of soluble factors, including paracrine factors, in a coculture system using Transwells. The data further suggested that when BM-MSCs were grafted into the injured liver, their roles could be improved in terms of liver function via paracrine effects of liver cytokines. However, further experiments are needed to clarify the underlying mechanisms mediating this phenomenon.

## Supplementary Material

Supplemental Material
