# Supplementary material for: Induction of the differentiation of porcine bone marrow mesenchymal stem cells into premature hepatocyte-like cells in an indirect coculture system with primary hepatocytes
Source: Anim Cells Syst (Seoul). 2020 Oct 4;24(5):289–98. doi: 10.1080/19768354.2020.1823473 (PMC7646558; doi:10.1080/19768354.2020.1823473)
Supplement: Supplemental Material [file TACS_A_1823473_SM0754.docx]

| Name | Sequence (5’→3’) | | Product size | Accession number |
| --- | --- | --- | --- | --- |
| *Albumin (ALB)* | Forward | GAAGACACCAGTGAGCGAAAAA | 69 | NM_001005208.1 |
|  | Reverse | AAGCAAGGCCGTCTGTTCA |  |  |
| *alpha Fetoprotein (AFP)* | Forward | CGTGCCTCCGCCATTC | 61 | NM_214317.1 |
|  | Reverse | CTGAGCTTGGCACAGATCCTT |  |  |
| *Transferrin (TF)* | Forward | CCGAAGACTGTATCGCCAAGA | 64 | X12386.1 |
|  | Reverse | TAGCCTCCATCCAAGCTCATG |  |  |
| *CYP3A29* | Forward | AGGAGGAGATTGAGGCAACTTTC | 63 | NM_214423.1 |
|  | Reverse | TCTGTGCCAGGGCATCGTA |  |  |
| *BAK* | Forward | TCA CCC TGC CCC TAG AAC CT | 101 | XM_001928147.2 |
|  | Reverse | CTG GAA TTC CGA GTC GTA TCG |  |  |
| *BCL2L1* | Forward | GGT ACC GGA GGG CAT TCA GT | 100 | NM_214285.1 |
|  | Reverse | TCC CGG AAG AGT TCG TTC AA |  |  |
| *GAPDH* | Forward | CCA TCT TCC AGG AGC GAG ATC | 100 | NM_001206359.1 |
|  | Reverse | GCC TTC TCC ATG GTC GTG AA |  |  |

Table 1. Primers used for real time PCR
